# A bacteriophage cocktail delivered in feed significantly reduced *Salmonella* colonization in challenged broiler chickens

**DOI:** 10.1080/22221751.2023.2217947

**Published:** 2023-06-20

**Authors:** Anisha M. Thanki, Steven Hooton, Natasha Whenham, Michael G. Salter, Mike R. Bedford, Helen V. Masey O'Neill, Martha R. J. Clokie

**Affiliations:** a Department of Genetics and Genome Biology, University of Leicester, Leicester, UK; b Ab Agri, Innovation Way, Peterborough Business Park, Peterborough, UK; c Ab Vista, Woodstock Court, Marlborough, UK

**Keywords:** *Salmonella*, phages, phage therapy, antimicrobial resistance, poultry

## Abstract

Nontyphoidal *Salmonella* spp. are a leading cause of human gastrointestinal infections and are commonly transmitted via the consumption of contaminated meat. To limit the spread of *Salmonella* and other food-borne pathogens in the food chain, bacteriophage (phage) therapy could be used during rearing or pre-harvest stages of animal production. This study was conducted to determine if a phage cocktail delivered in feed is capable of reducing *Salmonella* colonization in experimentally challenged chickens and to determine the optimal phage dose. A total of 672 broilers were divided into six treatment groups T1 (no phage diet and unchallenged); T2 (phage diet 10^6^ PFU/day); T3 (challenged group); T4 (phage diet 10^5^ PFU/day and challenged); T5 (phage diet 10^6^ PFU/day and challenged); and T6 (phage diet 10^7^ PFU/day and challenged). The liquid phage cocktail was added to mash diet with *ad libitum* access available throughout the study. By day 42 (the concluding day of the study), no *Salmonella* was detected in faecal samples collected from group T4. *Salmonella* was isolated from a small number of pens in groups T5 (3/16) and T6 (2/16) at ∼4 × 10^2^ CFU/g. In comparison, *Salmonella* was isolated from 7/16 pens in T3 at ∼3 × 10^4^ CFU/g. Phage treatment at all three doses had a positive impact on growth performance in challenged birds with increased weight gains in comparison to challenged birds with no phage diet. We showed delivering phages via feed was effective at reducing *Salmonella* colonization in chickens and our study highlights phages offer a promising tool to target bacterial infections in poultry.

## Introduction

Nontyphoidal *Salmonella* species are a global public health concern with ∼95.1 million human salmonellosis infections reported annually [1,2]. In the USA, the Centres of Disease Control and Prevention report over 1 million human cases of food-borne salmonellosis every year [3]; and in the European Union, 91,000 *Salmonella* infections are reported yearly [4]. Infections are frequently linked to consumption of animal-derived products especially poultry products (meat and eggs), which are common vehicles of *Salmonella* spp. transmission to humans. Chickens can become infected at any stage of the food chain from “farm to fork.” In a global effort to improve food safety, the poultry industry in the USA invests ∼$11.6 billion and the EU invests ∼€3 billion annually on multiple *Salmonella* preventive and control strategies including vaccination, antimicrobials, increased biosecurity, and competitive exclusion [5]. However, despite these strategies, *Salmonella* infections in poultry flocks remain high, continue to pose a constant food safety problem, and are a major contributor to morbidity and mortality of farm animals resulting in economic losses worldwide [3].

Antimicrobials are losing their effectiveness in treating bacterial diseases as pathogens are becoming resistant to frontline antibiotics and multidrug-resistant (MDR) pathogens are emerging in the poultry production chain [6]. The use of antibiotics is a necessity in large-scale animal production systems; however, poor stewardship via practices such as using antibiotics to promote growth has contributed to the emergence of antimicrobial resistance (AMR) [7,8]. Despite antibiotics being banned as growth promoters in countries including USA and in the EU, countries such as Brazil, China, and India continue to use antibiotics as growth promoters [9]. Selection for environmental AMR due to continued exposure to antimicrobials likely contributed to the spread of MDR strains within the food chain, including MDR *Salmonella* strains [10]. The prevalence of AMR in poultry products was recently reviewed which highlighted that *Salmonella* strains isolated from broiler chickens had high levels of resistance against common antibiotics used in poultry production. Levels of resistance against the antibiotic nalidixic acid (fluoroquinolone family) ranged from 43.6 to 97.6% and against ampicillin (β-lactam family) was 17.7 and 92.1% [11]. Worryingly, these MDR strains are entering the human food chain. Intervention strategies are needed to prevent and control the spread of *Salmonella* and other MDR bacteria in the food chain and bacteriophages (phages) could provide a solution [12].

Phages are viruses that infect and kill bacteria, are mostly bacterial species-specific, and can be used therapeutically to treat bacterial infections [13]. Several studies have shown phages are efficacious at reducing *Salmonella* colonization pre-harvest in challenged broiler birds or post-harvest by spraying carcasses or sliced chicken meat with phages [12,14]. A recent study showed mixing phage FGS011 into feed at dose 7.9 × 10^7^ plaque-forming units (PFU)/bird/day (feed was available *ad libitum* for 21 days) reduced *Salmonella* colonization from 54% in untreated *S. enteritidis*-challenged birds (*n *= 50) to 21% in challenged, phage fed birds (*n *= 50) after 6 weeks in collected caecal samples [15]. Similarly, a three phage cocktail delivered at 2 × 10^6^ PFU/bird/day in water reduced *Salmonella* counts by 1 log_10_ after 35 days (*n *= 60) in challenged broilers and significantly improved the performance of challenged birds, reducing the feed conversion ratio from 2.03 to 1.75 [16].

Similar to the studies discussed above, most challenge studies routinely test efficacy at one phage dose. However, it would be beneficial to test multiple doses to determine the optimal efficacious dose and safety of the product on the target animals at different doses as is conducted with antimicrobial compounds [17,18]. Such a phage dose response study would identify the minimum effective dose required to achieve significant therapeutic effects. Regulatory bodies may require dose response data when assessing efficacy and safety of phage products. Furthermore, obtaining phage dose response data increases the likelihood of identifying an economic optimum for delivery of treatments.

In this study, we tested the dose response of a phage cocktail (phages SPFM10-SPFM14) in a poultry *Salmonella* challenge trial. We used phages described previously that were collected from pig faeces and wastewater samples [19]. These phages are likely to be particularly efficacious in farm settings as our previous work has shown they can lyse MDR strains representative of serotypes *S.* Typhimurium, monophasic variants (*S*. 4:i:-, *S*. 4:i:-1,2, *S*. 4,12:i:- and *S*. 4,5,12:i:-), *S. Enteritidis, S. Infantis, S. Ohio*, *S*. 13,23:i:, *S*. Bovismorbifans and *S. Derby* [19,20]. We also previously showed a phage cocktail of SPFM10-SPFM14 can rapidly lyse target *Salmonella* strains both *in vitro* and *in vivo* using a *Galleria mellonella* larvae infection model [21]. The phage cocktail improved the survival of *Salmonella* challenged larvae from 3 to 85% after 72 h. Furthermore, we showed the phage cocktail administered prophylactically in feed at concentration 9.5 × 10^4^ PFU/g to challenged piglets significantly reduced *Salmonella* colonization in several gut compartments including in the colon and caecum [22].

As mentioned previously, limited data are available on how the number of phages administered to animals impacts disease prevention or treatment. To address this, we determined how phage dose impacts *Salmonella* colonization and performance of challenged birds. In this study, we tested the efficacy of phage cocktail SPFM10-SPFM14 delivered in feed at three doses: 10^5^, 10^6^, and 10^7^ PFU/day/chicken. The highest dose selected was considered on an optimum balance between commercial scale-up and phage efficacy.

## Materials and methods

### Strains and phages used in this study

The *Salmonella enterica* subsp. enterica serovar Typhimurium SL1344 strain (STSL1344) was used in this study for phage propagation and as the challenge strain in the chicken study. STSL1344 was grown on Xylose Lysine Deoxycholate (XLD) agar (Oxoid, UK) plates overnight at 37°C, on which *Salmonella* produced black colonies. To prepare liquid cultures, colonies were inoculated into Luria broth (LB) (Melford, UK) and grown overnight at 37°C at 100 rpm. Phages SPFM10 and SPFM14 were used in the present study, which are part of an international Leicester patent (filing date 24/09/2019 and number PCT/GB2019/052695) [19,21].

### Phage propagation and titration

Phages were propagated in liquid media as previously described [22]. Briefly, to exponentially growing liquid cultures of STSL1344 at concentration 10^7^ colony forming units (CFU)/mL, phages at titre 10^7^ PFU/mL were added. Bacterial and phage mix cultures were incubated at 37°C with shaking at 100 rpm for 6 h. After incubation samples were centrifuged for 15 min at 4200 × *g*, the supernatant was filtered using 0.22 µm pore size filters and stored at 4°C till use. Phage titres were determined by serially diluting lysates 10-fold in SM buffer (100 mM NaCl, 8 mM MgSO_4_·7H_2_O, and 50 mM Tris-Cl) and dilutions were plated via plaque assays [23,24] on LB 1% (w/v) agar plates with a lawn of STSL1344. Plates were then incubated overnight at 37°C.

### Basal and phage feed diet

Phages SPFM10 and SPFM14 were propagated individually as described above in LB and in total 7.5 L of the phage cocktail was produced at the University of Leicester. The final phage concentration of the cocktail with individual phages mixed at equal volumes was ∼3 × 10^11^ PFU/L and was used as the stock solution. The stock solution was diluted in SM buffer to achieve the three different phage doses. The required volumes of phage cocktail added to each phase diet are listed in Table 1 and were determined based on the average feed intake (FI) of chickens per diet phase. The phage and SM buffer liquid suspension were mixed into the mash starter (0–14 days), grower (14–28 days), and finisher diet (28–42 days). In the control feed diet in which phages were not added, SM buffer was added instead to ensure equal volumes of liquid of ∼2.1 L were added to each diet. The starter diet was manufactured 6 days prior to commencing the trial. During the trial, grower and finisher diets were manufactured 6 days prior to the change in diet. The diet compositions are listed in Supplementary Table 1. The diet without phage will be referred to as the basal diet and with phage as phage feed diet.

**Table 1.** Phage and buffer volumes added to starter, growth and finisher phage diets.

		Phase 1 starter (days 0–14)			Phase 2 grower (days 14–28)			Phase 3 finisher (days 28–42)		
Treatment group	Phage inclusion rate	Feed quantity (kg)	Phage inclusion rate (L)	Volume of buffer (L)	Feed quantity (kg)	Phage volume (L)	Volume of buffer (L)	Feed quantity (kg)	Phage inclusion rate (L)	Volume of buffer (L)
T1	0	75	0.000	2.143	225	0.000	1.940	375	0.000	1.856
T2	1×	75	0.214	1.929	225	0.194	1.746	375	0.186	1.670
T3	0	75	0.000	2.143	225	0.000	1.940	375	0.000	1.856
T4	0.1×	75	0.021	2.392	225	0.019	1.921	375	0.019	1.837
T5	1×	75	0.214	1.929	225	0.194	1.746	375	0.186	1.670
T6	10×	75	2.143	0.000	225	1.940	0.000	375	1.856	0.000

The titre of the phage cocktail in feed was tested 1 day after manufacture. Phage titres were determined by mixing 10 g of feed with 20 mL of SM buffer and the mix was rotated for 1 h. The mixture was subsequently centrifuged at 5000 × *g* for 20 min and the supernatant passaged through 0.22 µm pore size filters. Standard 10-fold serial dilutions in SM buffer were performed and plaque assays conducted to determine phage titres [24]. Final phage titres obtained in feed were expressed as PFU/g.

### Experimental design of challenge study

The experimental protocol was presented and accepted by Drayton Animal Health Welfare and the Ethical Review Body prior to the commencement of the study. The study was performed in accordance with the Animals (Scientific Procedures) Act 1986 and was conducted at Drayton Animal Health Ltd (UK). On study day (SD) 0, a total of 672 male Ross 208 broiler chicks were purchased from the commercial hatchery PD Hooks (UK). On arrival, the broilers were weighed and randomly assigned to one of six treatment groups. The six treatment groups comprised: T1 (unchallenged and fed basal diet), T2 (unchallenged and fed phage diet at dose 1× – 10^6^ PFU/day), T3 (challenged and fed basal diet), T4 (challenged and fed phage diet at dose 0.1× – 10^5^ PFU/day), T5 (challenged and fed phage diet at dose 1× – 10^6^ PFU/day), and T6 (challenged and fed phage diet at dose 10× – 10^7^ PFU/day) (Table 1 and Supplementary Table 1). Chicks were housed across 96 pens, with 7 chicks per pen across 5 environmentally controlled rooms (Supplementary Figure 1). Chicks allocated to pens (bedded with dust extracted wood shaving) had similar average weights per pen and there were 112 chicks per treatment group divided into 16 pens. All pens had free-access to feed-hoppers and water drinkers and both feed and water were provided *ad libitum*. On SD 0, the basal and phage feed diet was introduced to the relevant pens and the trial was conducted for 42 days (Supplementary Table 1). Chickens in each pen were weighed on SD 0, 14, 28, and 42. FI was recorded from SD 0 to 14, SD 14 to 28, SD 28 to 42, which was in accordance with feed phase changes. In addition, overall feed consumed from SD 0 to 42 for the study period was recorded.

In total, 448 chickens were divided equally into treatment groups T3, T4, T5, and T6 and were challenged with STSL1344 on SD 4 at a final dose of 10^6^ CFU/bird. The bacterial inoculum was prepared at the University of Leicester 1 h prior to challenge and transported on ice to the trial facility. The challenge strain was prepared by growing STSL1344 overnight in 10 mL LB broth at 37°C at 100 rpm, after which the culture was centrifuged at 4200 × *g* for 10 min and the supernatant was discarded. The pellet was resuspended in 10 mL PBS and the centrifuge step was repeated. The supernatant was discarded and the washed pellet was resuspended in 10 mL PBS, which was used as the challenge material. Chicks received 0.2 mL of the challenge by oral gavage using a blunt-ended cannula.

Composite faecal samples were taken from the floor of pens in each room to represent the different treatment groups on SD 2. Pooled faecal samples were collected from all 96 pens on SD’s 6, 7, 8, 9, 10, 14, 28, and 42. To facilitate faecal sampling, fresh cardboard was placed in each pen in the evening before sampling and once collected was placed on ice. Faecal samples were processed immediately after collection to determine *Salmonella* counts. Faecal material (1 g) was mixed with 9 mL PBS, vortexed for 5 s, and diluted 10-fold in PBS (undiluted to 10^−7^). 10 µL of each dilution were spot tested on XLD agar plates in triplicate, incubated overnight at 37°C and if *Salmonella* was present, the counts were expressed as CFU/g [22]. To determine phage counts from faecal samples, 1 g of faecal matter was mixed with 9 ml of SM buffer, centrifuged at 5000 × *g* for 20 min, filtered using 0.22 µm filters, and stored at 4°C till use. The filtrate was diluted 10-fold (undiluted to 10^−4^), all dilutions were plated via plaque assays and total phage counts were expressed as PFU/g [24].

On the final day of the trial on SD 42, two chickens per pen (192 chickens in total) were humanely euthanized by cervical dislocation, followed by exsanguination and were necropsied to collect caeca. Samples were stored on ice and processed immediately to determine *Salmonella* and phage counts (as described above). On SD 42 chickens in T1 were returned to the food chain following veterinary inspection. The remaining chickens in treatment groups T2, T3, T4, T5, and T6 were humanely euthanized by cervical dislocation, followed by exsanguination.

### Sensitivity of isolated Salmonella colonies during the chicken trial to the phage cocktail

If *Salmonella* colonies were isolated from faecal sampling, colonies were picked, streaked on XLD agar plates, and incubated overnight at 37°C. *Salmonella* colonies were re-streaked three times on XLD agar plates and plates were stored at 4°C prior to phage sensitivity testing. Putative resistant colonies were inoculated in LB broth and grown overnight at 37°C at 100 rpm. Sensitivity of putatively resistant STSL1344 cultures to phages SPFM10 and SPFM14 were tested. The phage stocks used were the original stocks added in phase diets at titres 10^8^ PFU/mL and individual phages were diluted 10-fold (undiluted to 10^−6^) [25]. Serial dilutions were plated via plaque assays on lawns prepared from cultures of the putatively resistant colonies and phage counts expressed as PFU/mL. Efficiency of plating (EOP) was compared to the wild-type strain STSL1344 to determine if there was a difference in killing efficiency.

### Statistical analysis

Each pen was considered as an experimental unit and for each treatment group there were 16 replicates. To determine if there were significant differences in *Salmonella* and phage counts between treatment groups, student *T*-tests were conducted and *p* values <0.05 were considered as significant using the program Prism 9 version 9.0.2 (134). For performance data on body weight, body weight gain, FI, feed conversion ratio (FCR) and mortality corrected FCR analysis of variance was conducted using the analytical software JMP Pro 14 and *p* values < 0.05 were considered as significant. Kruskal–Wallis analysis was conducted for chicken mortality.

## Results

### Phage recovery from feed

**Figure 1.**
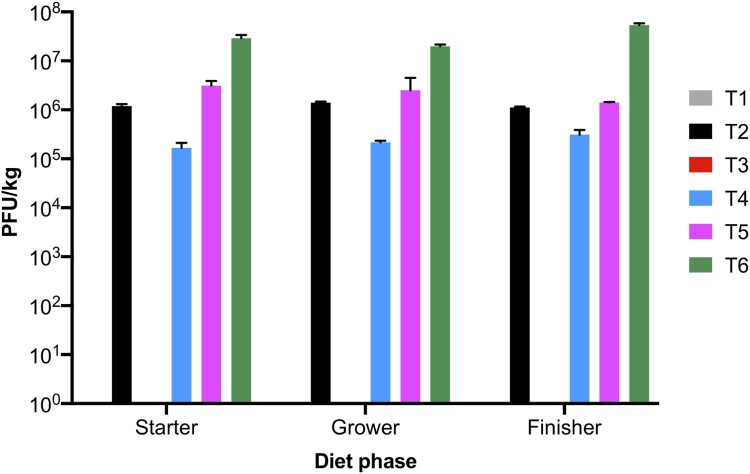
Phage titres in starter, grower, and finisher mash diets. Three technical replicates were conducted for each diet and the error bars show standard error of the mean.

The phage cocktail with phages SPFM10 and SPFM14 was added to starter (0–14 days), grower (14–28 days), and finisher (28–42 days) phase diets produced for groups T2 (10^6^ PFU/kg), T4 (10^5^ PFU/kg), T5 (10^6^ PFU/kg), and T6 (10^7^ PFU/kg). The phage cocktail in feed was titred after phase diets were manufactured and average phage titres across all three phases were 1.2 × 10^6^ PFU/kg (T2), 3.0 × 10^5^ PFU/kg (T4), 2.4 × 10^6^ PFU/kg (T5), and 3.4 × 10^7^ PFU/kg (T6) (Figure 1). The phage titres in feed were stable, with no loss in titres. As expected, no phages were recovered from all basal diets produced for groups T1 and T3, in which no phage cocktail was added.

### Challenge chicken study

#### Composite faecal samples

Individual composite faecal samples were taken from pens in each treatment group on SD 2 prior to STSL1344 challenge and no *Salmonella* were isolated indicating birds were not infected with *Salmonella* prior to commencing the study.

#### Salmonella counts in intestinal faecal samples

**Figure 2.**
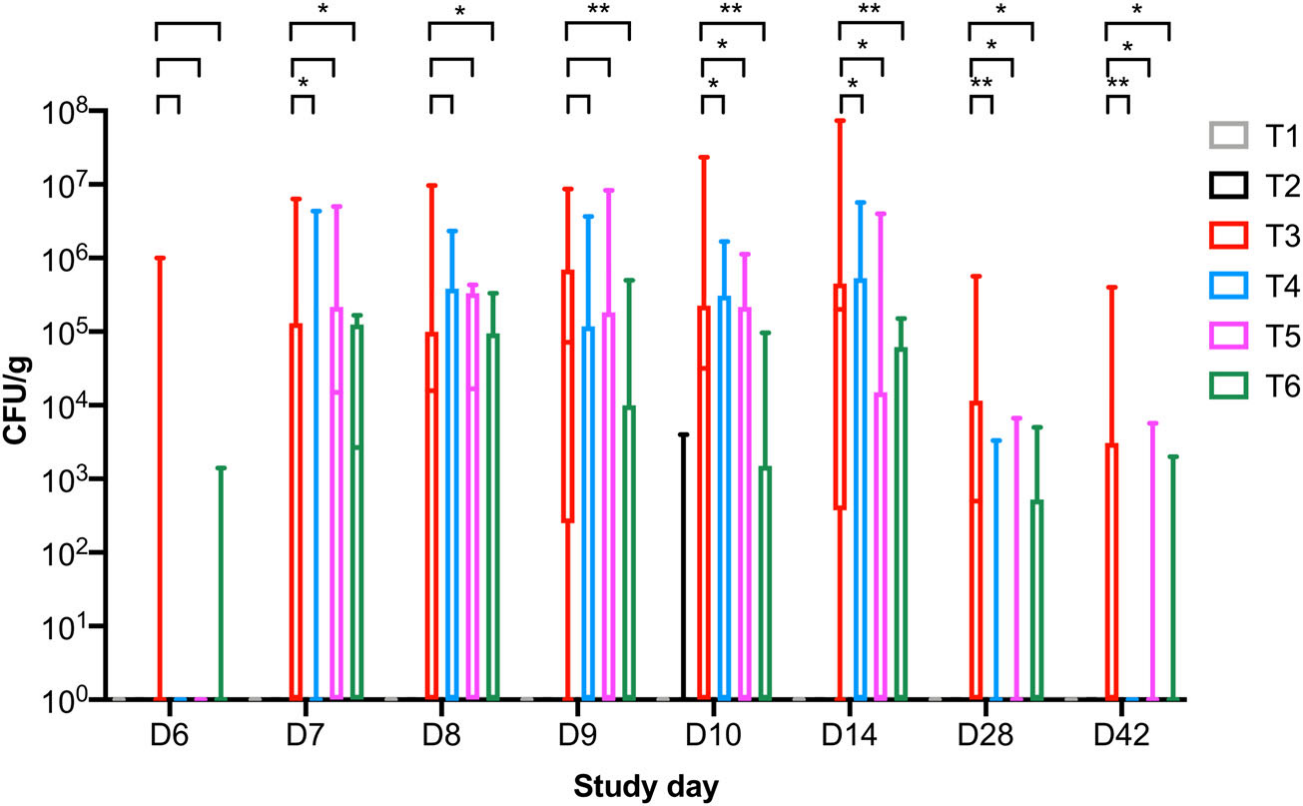
Boxplot shows the maximum and minimum *Salmonella* counts isolated from faecal samples collected from the six treatment groups. *Salmonella* counts in challenged group T3 were compared to challenged and phage-treated groups T4 (10^5^ PFU/day), T5 (10^6^ PFU/day), and T6 (10^7^ PFU/day). Statistical differences between treatment groups (*n* = 16) are displayed on the graph (*p < 0.05 and **p < 0.01).

**Figure 3.**
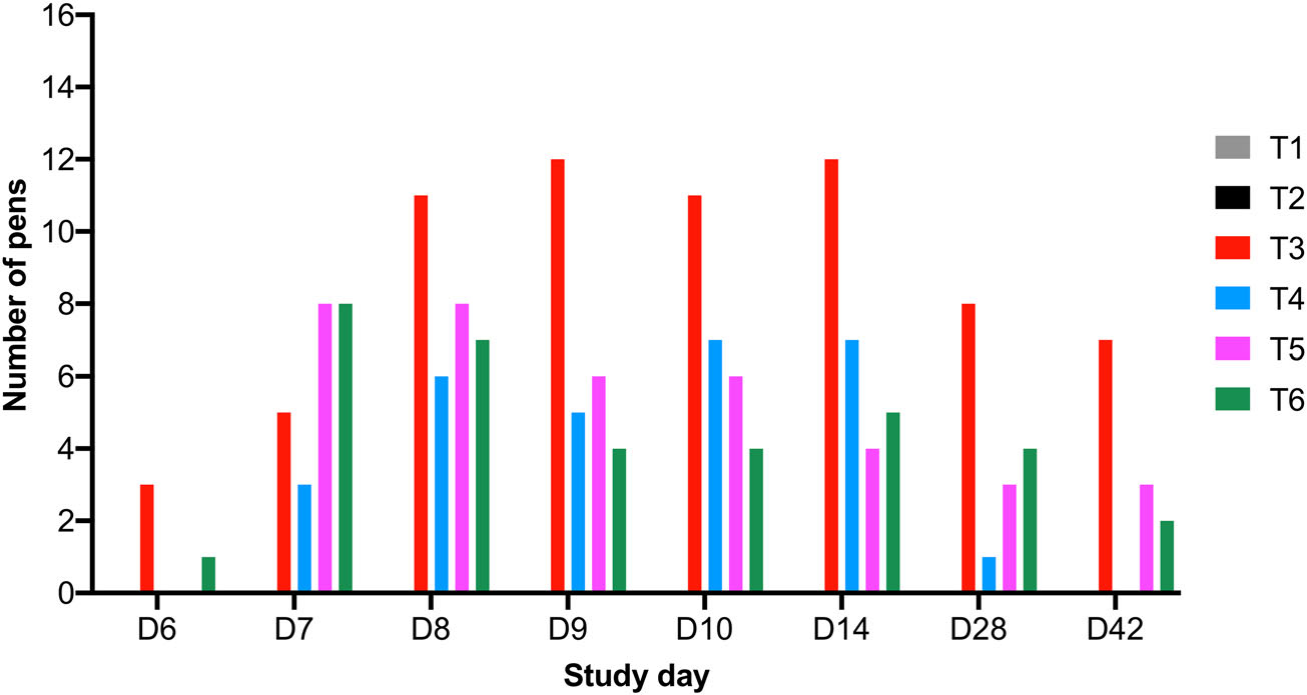
Graph shows the number of pens from which *Salmonella* was isolated from faecal samples over the course of the study and there were 16 pens per treatment group.

*Salmonella* counts were determined from faecal samples collected over the course of the trial and *Salmonella* was only reisolated from challenged groups T3, T4, T5, and T6. The first faecal sampling point was 2 days post-challenge on SD 6 and *Salmonella* was isolated from 3/16 and 1/16 pens in groups T3 and T6, respectively (Supplementary Table 2, Figures 2 and 3). In samples collected from phage-treated groups T4 and T5, *Salmonella* was not isolated from faecal samples, suggesting birds were not colonized with *Salmonella* at this timepoint. On SD 7, *Salmonella* was isolated from challenged groups and was detected in 50% of pens for groups T5 and T6. In samples collected from T4, *Salmonella* was detected in 3/16 pens and in T3 from 5/16 pens.

On SD 8, the average *Salmonella* counts were higher in groups T3, T4, and T5 and were ∼6.0 × 10^5^ CFU/g (*p* > 0.05) and *Salmonella* was isolated from 11/16 pens in group T3 (Supplementary Table 2 and Figure 2). However, *Salmonella* was isolated from 6/16 and 8/16 pens for groups T4 and T5, respectively. In group T6, average counts were significantly lower at 7.04 × 10^4^ CFU/g (*p* < 0.05). Similar counts were observed from collected samples on SD 9. On SD’s 10 and 14, the average *Salmonella* counts increased in group T3 to ∼3 × 10^6^ CFU/g and *Salmonella* was isolated from 12/16 pens. However, in all phage treatment groups, the counts were significantly lower (*p* < 0.05). On SD 10, *Salmonella* was isolated from 7, 6, and 4/16 pens from groups T4, T5, and T6, respectively. On SD 14, *Salmonella* was isolated from 7, 4, and 5/16 pens from groups T4, T5, and T6, respectively (Figure 3).

On SD 28, the average *Salmonella* counts in challenged chickens in group T3 were 5.73 × 10^4^ CFU/g and *Salmonella* was isolated from faecal samples collected from 8/16 pens. In comparison, there were significant reductions in *Salmonella* counts in all phage treatment groups (*p* < 0.05) and *Salmonella* was isolated from 1, 3 and 4/16 pens from groups T4, T5, and T6, respectively. On SD 42 *Salmonella* was isolated from 0, 3, and 2/16 pens from groups T4, T5, and T6, respectively, and *Salmonella* was isolated from 7/16 from group T3. The average bacterial counts for group T3 were 3.06 × 10^4^ CFU/g and all three phage doses significantly reduced *Salmonella* counts to 0.00, 6.46 × 10^2^ and 1.88 × 10^2^ CFU/g (*p* < 0.05) for groups T4, T5, and T6, respectively (Supplementary Table 2, Figures 2 and 3). In terms of *Salmonella* clearance the lowest phage dose tested was the most effective.

#### Phage counts in intestinal faecal samples

**Figure 4.**
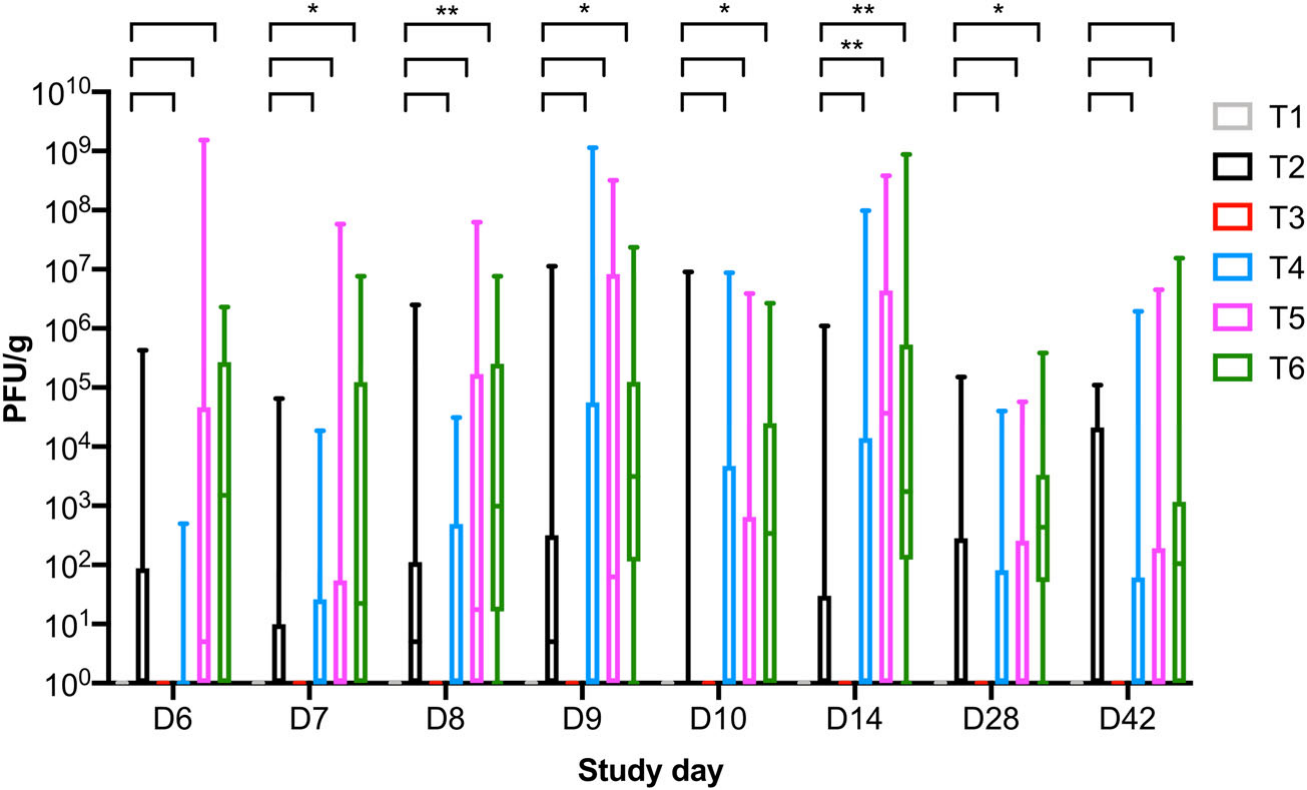
Boxplot shows the maximum and minimum phage counts isolated from faecal samples collected from the six treatment groups. Phages were reisolated from all phage-treated groups: T2 bird fed phage at dose 10^6^ PFU/day; and challenged and fed the phage diet groups T4 (10^5^ PFU/day), T5 (10^6^ PFU/day), and T6 (10^7^ PFU/day).

From the collected faecal samples, total phage counts were also determined (Figure 4). From all eight sampling points, phages were not isolated from treatment groups that were not fed the phage diet (T1 and T3). T2 was fed the 1× phage dose mixed with the mash diet and challenged groups T4, T5, and T6 were fed the 0.1×, 1×, and 10× phage doses, respectively. Groups T2 and T5 were fed the same dose diet.

On SD 6, 2 days after challenge the total phage cocktail counts were on average 2.75 × 10^4^, 3.16 × 10^1^, 9.76 × 10^7^, and 3.25 × 10^5^ PFU/g for groups T2, T4, T5, and T6, respectively (Figure 3). The phage counts show there was phage multiplication in T5 when the counts for T2 were compared to T5, as average phage counts were 1000 times higher in faecal samples collected from T5. A similar trend in phage counts was observed on SD 7 and SD 8 for groups T2 and T5. Phage counts for treatment groups T4 and T6 on SD 7 and SD 8 were similar to SD 6. On SD 9, the average phage counts were higher for all challenged phage-treated groups and the counts were 7.13 × 10^7^, 3.12 × 10^7^, and 1.56 × 10^6^ for groups T4, T5, and T6, respectively. In comparison, the average phage counts were 7.17 × 10^5^ PFU/g for group T2.

On SD 10, the average counts were similar for all phage groups. On SD 14, phage counts were higher for all challenged phage treatment groups by 2–3 log10 PFU/g in comparison to group T2. This again suggests phage replication and multiplication was occurring in the phage-treated groups. On SD 28, phage counts were similar across groups T2, T4, and T5 at 6 × 10^3^ PFU/g and the counts were significantly higher in T6 samples at 4 × 10^4^ PFU/g. On SD 42, the average phage counts were similar across all phage groups.

#### Analysis of caeca samples

**Figure 5.**
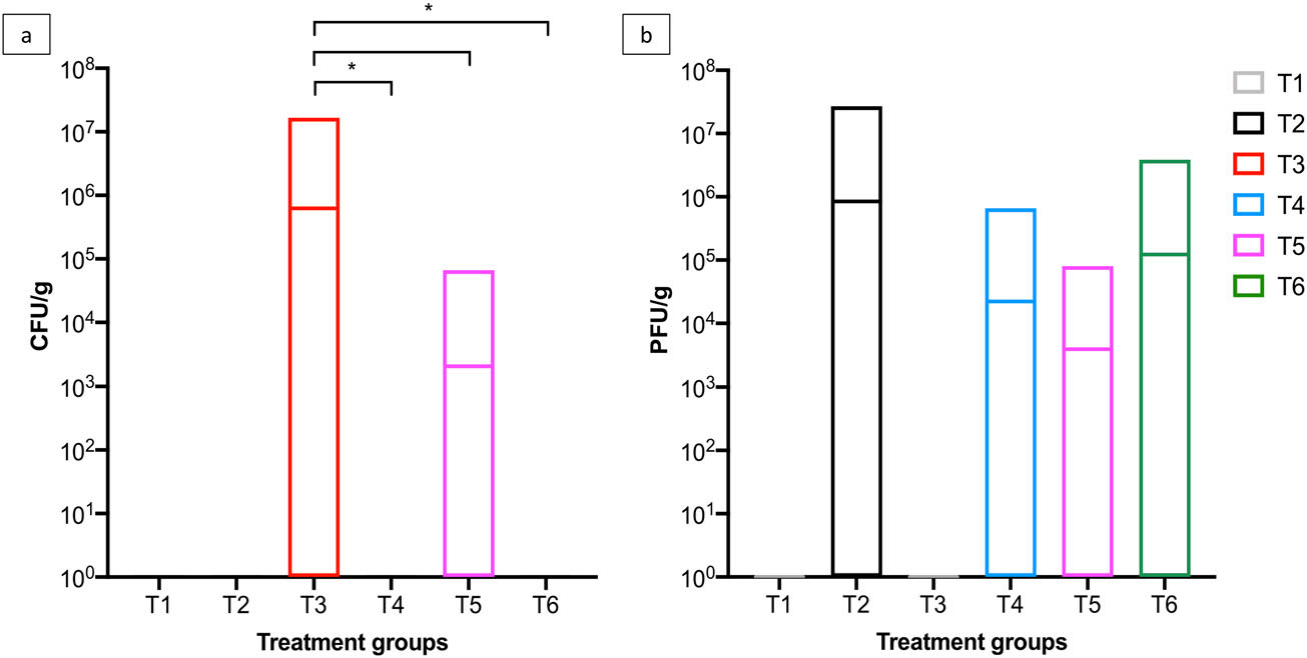
Boxplots show the (a) *Salmonella* and (b) phage counts isolated from caeca samples collected on SD 42, the last day of the study. *Salmonella* counts in challenged group T3 were compared to challenged and phage-treated groups T4 (10^5^ PFU/day), T5 (10^6^ PFU/day), and T6 (10^7^ PFU/day). Statistically differences between treatment groups are displayed on the graph (*p < 0.05).

On SD 42, two chickens from each pen were euthanized and their caeca was removed to determine *Salmonella* and phage counts. *Salmonella* was not isolated from caeca samples collected from unchallenged chickens in groups T1 and T2. In samples collected from groups T3 and T5, *Salmonella* was isolated from 20/32 and 1/32 caeca samples and the average counts were 6.27 × 10^5^ CFU/g and 2.08 × 10^3^ CFU/g, respectively. *Salmonella* was not isolated from caeca samples collected from groups T4 and T6. Phages were isolated from all phage treatment groups (T2, T4, T5, and T6) and T2 caeca samples had the highest average phage count of 8.44 × 10^5^ PFU/g. In caeca samples from groups T4, T5, and T6, the total phage counts were 2.23 × 10^4^, 3.98 × 10^3^, and 1.22 × 10^5^ PFU/g (Figure 5).

#### Performance data

In Table 2, the performance data are categorised as phase 1 (SD’s 0–14), phase 2 (SD’s 14–28), and phase 3 (SD’s 28–42) to reflect the changes in phase diets of starter, grower, and finisher diets, respectively. In addition, the table includes overall performance scores for SD's 0–42. The performance parameters analysed were mean body weight (BW), body weight gain, FI, feed conversion ratio (FCR), mortality and mortality corrected feed conversion ratio (mFCR).

**Table 2.** Performance data and different letters donate significant differences between groups at *p* < 0.05.

Treatment group		T1	T2	T3	T4	T5	T6	SEM	*P* value
**Challenge**	** **	No	No	Yes	Yes	Yes	Yes	** **	** **
**Phage dose**	** **	0	1×	0	0.1×	1×	10×	** **	** **
**Day 0**	**Mean BW (kg)**	0.0424^a^	0.0428^a^	0.0421^ab^	0.0415^b^	0.0413^b^	0.0414^b^	0.00014	0.0033
**Day 14**	**Mean BW (kg)**	0.410	0.390	0.409	0.403	0.410	0.405	0.00259	0.2227
**Day 28**	**Mean BW (kg)**	1.435^a^	1.358^b^	1.339^b^	1.353^b^	1.377^ab^	1.417^a^	0.00899	0.0082
**Day 42**	**Mean BW (kg)**	2.817^a^	2.642^bc^	2.605^c^	2.642^bc^	2.620^bc^	2.713^b^	0.0154	0.0002
**Days 0–14**	**Gain (kg)**	0.367	0.348	0.367	0.362	0.369	0.363	0.00259	0.1768
	**FI (g/bird/day)**	41.88^a^	36.06^d^	37.20^cd^	40.68^ab^	38.21^bcd^	39.07^bc^	0.00297	0.0004
	**FCR**	1.60	1.45	1.51	1.56	1.51	1.54	0.0210	0.4593
	**Mortality (%)**	0.000	0.000	0.893	0.000	0.000	1.786	0.2499	0.2097
	**mFCR**	1.60	1.45	1.49	1.56	1.51	1.50	0.0208	0.3900
**Days 14–28**	**Gain (kg)**	1.025^a^	0.968^bc^	0.942^c^	0.950^c^	0.973^bc^	1.012^ab^	0.00730	0.0021
	**FI (g/bird/day)**	110.80^a^	103.36^bc^	100.50^c^	101.67^bc^	103.10^bc^	105.99^ab^	0.7785	0.0011
	**FCR**	1.48	1.51	1.49	1.50	1.47	1.47	0.00731	0.5205
	**Mortality (%)**	0.000	0.893	0.893	0.000	0.000	0.000	0.2052	0.5433
	**mFCR**	1.48	1.50	1.49	1.50	1.47	1.47	0.00705	0.5603
**Days 28–42**	**Gain (kg)**	1.383^a^	1.284^bc^	1.265^bc^	1.289^bc^	1.245^c^	1.315^b^	0.00886	<0.001
	**FI (g/bird/day)**	182.42^a^	170.90^b^	170.72^b^	171.17^b^	173.70^b^	175.11^ab^	1.1470	0.0212
	**FCR**	1.83^c^	1.86^bc^	1.90^b^	1.86^bc^	1.95^a^	1.85^bc^	0.00936	0.0009
	**Mortality (%)**	0.000	0.000	0.893	0.000	0.000	0.000	0.1458	0.4159
	**mFCR**	1.83^b^	1.86^b^	1.88^b^	1.86^b^	1.95^a^	1.85^b^	0.00919	0.0009
**Days 0–42**	**Gain (kg)**	2.775^a^	2.599^bc^	2.563^c^	2.600^bc^	2.581^bc^	2.672^b^	0.0153	0.0002
	**FI (g/bird/day)**	111.70^a^	102.51^bc^	102.15^c^	104.46^bc^	105.04^bc^	106.44^b^	0.6701	0.0002
	**FCR**	1.69	1.66	1.70	1.69	1.70	1.67	0.00767	0.6105
	**Mortality (%)**	0.000	0.893	2.679	0.000	0.000	1.786	0.3477	0.1334
	**mFCR**	1.69	1.66	1.70	1.69	1.70	1.67	0.00767	0.6105

SEM is the standard error of the mean.

During the first phase on SD 0, there were significant differences in body weight gain between treatment groups (*p* = 0.0033). At SD 14, there was no significant difference in the mean body weight between treatment groups. On SD 28 groups T1 and T6 had the highest mean body weight of 1.435 and 1.417 kg, respectively, followed by group T5. Groups T2, T3, T4 had the lowest mean weight (*p* = 0.0082). On SD 42, there were significant differences between the groups (*p* = 0.0002) and T1 and T6 again had the highest mean body weight and T3 had the lowest at 2.605 kg (Table 2).

Chickens were challenged during phase 1 (SD's 0–14) but there was no difference in weight gain, FCR, mortality, and mFCR between the treatment groups over this period. FI varied between the groups and overall chickens in T1 consumed the most at 41.88 g/bird/day (*p* = 0.0004). Chickens in groups T4 and T6 had the second highest FI.

During the second phase, daily weight gain and FI were significantly different between the treatment groups (*p* = 0.0021). Chickens in groups T1 and T6 gained the most weight and consumed the most food. The data shows a dose dependent response based on the phage cocktail dose as weight gain and FI increased as the phage dose in feed was increased by 10-fold in groups T4, T5, and T6. In comparison, challenged chickens fed the basal diet in group T3 had the lowest weight gain and FI. FCR, mortality, and mFCR were similar for all treatment groups.

During the third phase mortality did not differ between the treatment groups. However, weight gain, FI, FCR, and mFCR were significantly different (*p* < 0.001, *p* = 0.212, *p* = 0.0009 and *p* = 0.0009, respectively). Again, weight gain was highest for group T1 and weights were similar for groups T2, T3, and T4. Chickens in group T5 had the lowest weight gain. There was a phage dose dependent increase in FI in groups T4, T5 and T6 of 171.17, 173.70 and 175.11 g/bird/day. FCR and mFCR values were lowest for T1 followed by T2, T4, and T6. Groups T3 and T5 had the highest FCR.

Over the course of the trial from SD 0 to 42 T1 had the highest average weight gain at 2.775 kg, FI of 111.70 g/bird/day, and the best performance (*p* = 0.0002). Performance of birds in group T6 was statistically the second best with weight gain at 2.672 kg and FI 106.44 g/bird/day (Table 2). Phage-treated groups T2, T4, and T5 followed third and birds in the challenge-only group T3 had the lowest performance. Therefore, phage treatment did improve the performance of challenged birds. Despite the differences in weight gain and FI, FCR, mortality, and mFCR did not significantly differ between the treatment groups.

#### Phage susceptibility of reisolated Salmonella colonies

**Figure 6.**
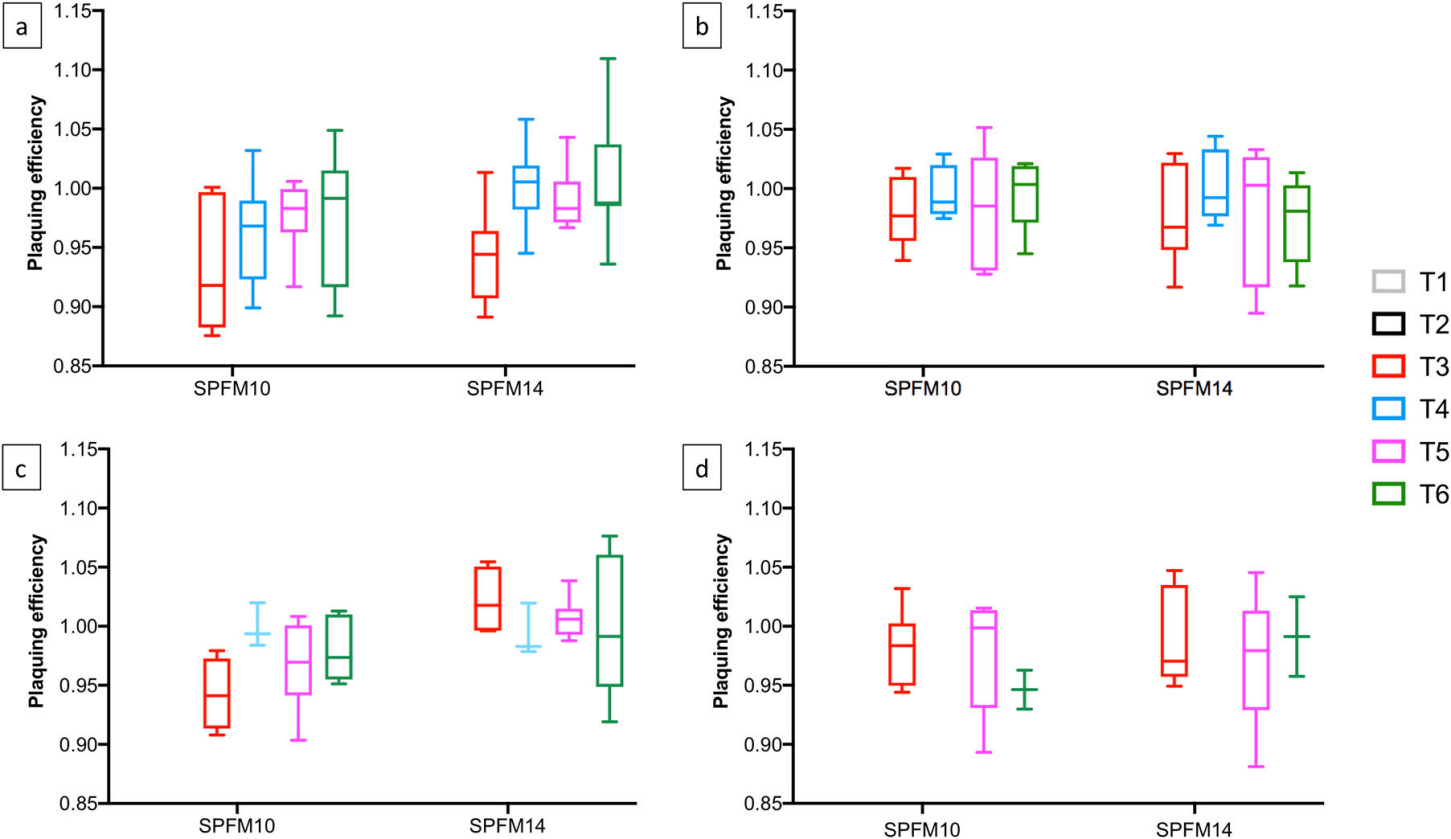
Graphs show the EOP of phages SPFM10 and SPFM14 on *Salmonella* colonies isolated from faecal samples collected on SD 10 (a), SD 14 (b), SD 28 (c), and SD 42 (d) over the course of the trial.

Resistance studies were conducted to determine if *Salmonella* colonies reisolated from faecal samples collected from challenged groups remained sensitive to the phages within the cocktail. *Salmonella* colonies isolated from group T3 were also included in the study to determine if the wild-type strain, which has not been exposed to the phage cocktail, remained phage sensitive. For the study *Salmonella* colonies isolated on SD’s 10, 14, 28, and 42 from faecal sampling were screened and the EOP of phages SPFM10 and SPFM14 were compared to their EOP on the wild-type strain. Overall, across the four sampling days the EOP of both phages were not significantly different (*p* < 0.05) to their EOP on the wild-type strain, which suggests phage resistance did not build up over the course of the trial (Figure 6).

## Discussion

Phage therapy has gained momentum in recent years and offers a viable alternative to antibiotics to reduce the presence of *Salmonella* in the human food chain. The poultry production environment presents a considerable challenge for controlling AMR and phages have been shown to effectively control MDR bacteria. Further data including trial phage efficacy data in challenged chickens, optimal dosage requirements for achieving therapeutic efficacy, and data examining the potential development of phage resistance is required for the development of a robust phage cocktail product. In this study we collected this relevant data by conducting a large-scale *Salmonella* challenge chicken study, in which we investigated the efficacy of a phage cocktail reducing *Salmonella* colonization at three separate doses.

The phage cocktail was delivered to chickens by mixing liquid phages into the mash diet of starter, grower, and finisher feed and the phage cocktail was stable within the diet. Adding the phage cocktail after manufacturing the diet was necessary as heat and high pressures used during the production process could affect phage viability, especially phages in liquid [26]. Commercially, this could be a viable option for phage delivery in feed as additives are currently being added to final feed products, such as by mixing with or spraying on feed. Other potential delivery options could be drying the phages with excipients, which protect during the drying process and then mixing the dried phages with feed and pelleting at commercial scale [22]. We have recently shown delivering the same phage cocktail as used in the current study via this route is viable but as the drying excipients tested are expensive it would add extra costs to the manufacturing [27].

All three phage cocktail doses (0.1×, 1×, and 10×) tested significantly reduced *Salmonella* colonization in challenged chickens by SD 10, which was only 6 days after challenge and counts remained lower until the end of the trial on SD 42 in comparison to challenged birds in group T3. Furthermore, by SD 8, *Salmonella* was consistently isolated from fewer pens in all phage-treated groups in comparison to T3, which highlights the effectiveness of phage treatment. A possible explanation could be that administering the phage cocktail prophylactically could have prevented the establishment of infection as studies have shown phages can line intestinal epithelial cells, and thus could limit adherence of fewer *Salmonella* cells [28,29]. In another study, Bardina et al. showed orally administering a phage cocktail 24 h prior to challenge and then frequently after challenge significantly reduced *Salmonella* colonization in caeca [30]. Similarly, Nabil et al. found administering phage one day prior to challenge caused significant reduction in *Salmonella* counts in the caeca five days post-challenge [31]. These studies, alongside the data presented here, provide convincing evidence that prophylactic phage treatment is an effective approach to reducing *Salmonella* colonization in chickens.

Based on the *Salmonella* counts isolated from faecal samples, initially the highest phage dose of 10× (group T6) was most effective with significant reductions in *Salmonella* colonization being observed 3 days post-challenge. Furthermore, until SD 14, *Salmonella* was isolated from fewer pens in group T6 in comparison to birds in group T4 fed the lowest phage cocktail dose. However, when comparing the phage-treated groups at SD 28, group T4 had the lowest *Salmonella* counts in faecal samples and *Salmonella* was only isolated from one out of 16 pens. At SD 42 *Salmonella* was not isolated from faecal and caecal samples collected from T4 pens, which indicates complete clearance of infection and shows this phage dose was the most effective. In comparison in groups T5 and T6, *Salmonella* was isolated from three and two pens, respectively. Nevertheless, the average *Salmonella* counts and the number of pens it was isolated from were still significantly lower than group T3. The data shows all three doses were effective at reducing *Salmonella* colonization in chickens. However, based on the *Salmonella* clearance data from SD 42, the 10^5^ PFU/day dose was most effective and reduced counts to below detection levels. The reason for the lower dose being the most effective is unknown. However, we speculate it may be related to this dose having a lower rate of bacterial killing which was observed till SD 28. This lower rate of bacterial killing could allow for increased levels of localized infection and replication before its clearance by the host immune system.

Data on optimal phage dose are limited and to our knowledge there is only one other study which has investigated phage dose in a *Salmonella* chicken challenge study. Lim et al. tested the efficacy of phage doses 10^5^ (0.01×), 10^7^ (1×), and 10^9^ (100×) PFU/g and to compare the doses to the present study, the doses in brackets are shown in the same format as used in our study. They showed the higher phage doses of 10^7^ (1×) and 10^9^ (100×) PFU/g, were the only doses to cause significant reductions in *S. Enteritidis* colonization 3 weeks after challenge and the lowest dose 10^5^ (0.01×) PFU/g had no effect. The higher phage doses reduced *Salmonella* counts by less than 1 × 10^1^ CFU/g in comparison to challenged birds [32]. However, in the present study, our phage cocktail caused higher reductions of ∼2 × 10^2^ CFU/g at doses 1× and 10× at SD 42, even though the highest dose was lower than the one used in the Lim study, which highlights the effectiveness of the cocktail we have developed.

The performance data based on weight gain and FI over 42 days highlighted all three doses of phage treatment, especially the 10× dose significantly improved the bird’s performance in comparison to challenge-only birds, who overall had the lowest performance. In addition, the performance data importantly showed phage treatment was not having a negative impact on performance, thus is safe for use in chickens and has also been highlighted in other challenge studies [12]. *Salmonella* contamination, therefore, not only contaminates the end product, which is of a human health concern, but also reduces performance of birds as well. Thus, phage treatment could well have two benefits for the poultry industry, the first relating to decontamination of poultry products and the second being increased commercial return for the producer.

In summary, our study investigated different phage cocktail doses to determine which dose reduces *Salmonella* colonization in chickens most efficiently. We found that the lowest phage dose was effective at reducing *Salmonella* colonization by the end of trial, but birds given the highest phage dose performed better. Future research will focus on investigating safety of phage therapy by determining the impact of phages on the microbiome of chickens and use histopathology to determine pathological impact (if any) of phage infection in chickens organs.

## Supplementary Material

Supplemental Material
