# Supplementary material for: A bacteriophage cocktail delivered in feed significantly reduced Salmonella colonization in challenged broiler chickens
Source: Emerg Microbes Infect. 2023 Jun 20;12(1):2217947. doi: 10.1080/22221751.2023.2217947 (PMC10283443; doi:10.1080/22221751.2023.2217947)
Supplement: Supplemental Material [file TEMI_A_2217947_SM5946.docx]

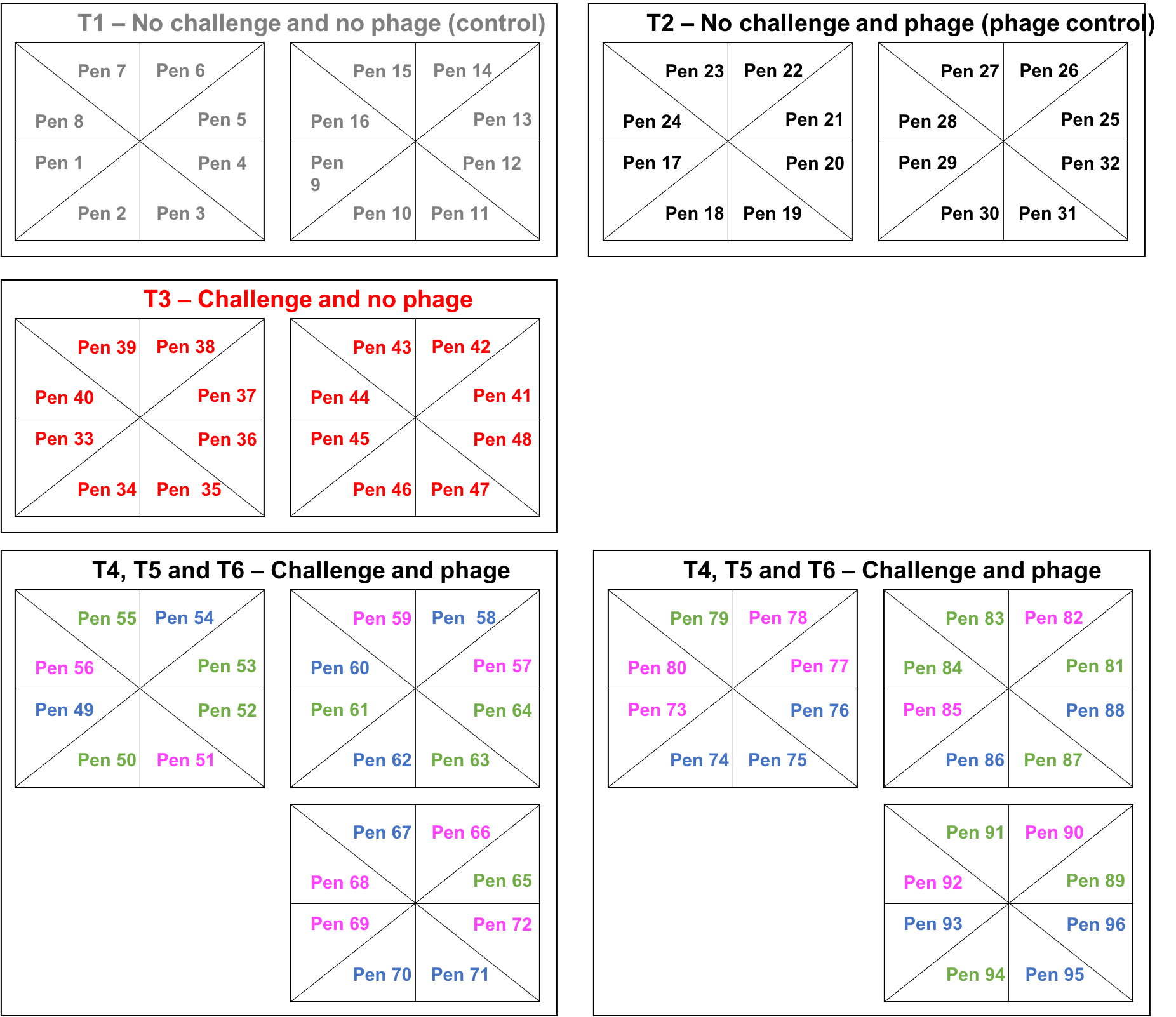


**Supplementary Figure 1**. Chickens were housed across five rooms dependent on the treatment groups and images shows the pen layout. Challenged and phage groups T4, T5 and T6 were grouped across two rooms. Challenged birds given phage dose 10^5^ PFU/day, 10^6^ PFU/day and 10^7^ PFU/day and shown in blue, pink and green text respectively.

**Supplementary Table 1**. Diet formulations used in starter, grower and finisher mash diets.

| **Ingredient** | **Starter**  **Days 0-14** | **Grower**  **Days 15-28** | **Finisher**  **Days 29-42** |
| --- | --- | --- | --- |
| **Wheat - Feed** | 56.41% | 63.71% | 71.47% |
| **Soybean meal 48** | 38.64% | 31.19% | 22.62% |
| **Soy oil** | 1.99% | 2.77% | 3.67% |
| **Salt** | 0.35% | 0.35% | 0.35% |
| **DL Methionine** | 0.24% | 0.17% | 0.18% |
| **Lysine HCl** | 0.09% | 0.09% | 0.14% |
| **Limestone** | 0.89% | 0.82% | 0.83% |
| **Dicalcium Phos** | 0.85% | 0.36% | 0.21% |
| **Quantum Blue** | 0.01% | 0.01% | 0.01% |
| **Econase** | 0.01% | 0.01% | 0.01% |
| **Vitamin premix** | 0.50% | 0.50% | 0.50% |
| **Crude protein %** | 24.71 | 21.88 | 18.64 |
| **Poult ME kcal/kg** | 3,000.00 | 3,100.00 | 3,200.00 |
| **Pig DE Kcal** | 3,447.61 | 3,470.22 | 3,480.76 |
| **Calcium %** | 0.96 | 0.80 | 0.75 |
| **Phos %** | 0.78 | 0.65 | 0.59 |
| **Avail Phos %** | 0.48 | 0.39 | 0.36 |
| **Fat %** | 3.40 | 4.19 | 5.10 |
| **Fibre %** | 2.70 | 2.62 | 2.52 |
| **Met %** | 0.59 | 0.49 | 0.45 |
| **Cys %** | 0.42 | 0.38 | 0.34 |
| **Me+Cys %** | 1.01 | 0.87 | 0.79 |
| **Lys %** | 1.40 | 1.20 | 1.00 |
| **His %** | 0.62 | 0.55 | 0.46 |
| **Tryp %** | 0.31 | 0.28 | 0.23 |
| **Thr %** | 0.91 | 0.79 | 0.65 |
| **Arg %** | 1.65 | 1.42 | 1.15 |
| **Iso %** | 1.04 | 0.90 | 0.74 |
| **Leu %** | 1.84 | 1.62 | 1.35 |
| **Phe %** | 1.18 | 1.04 | 0.87 |
| **Tyr %** | 0.84 | 0.73 | 0.61 |
| **Val %** | 1.12 | 0.99 | 0.83 |
| **Gly %** | 1.02 | 0.90 | 0.77 |
| **Ser %** | 1.18 | 1.04 | 0.88 |
| **Phe+Tyr %** | 2.02 | 1.77 | 1.48 |
| **D Met%** | 0.53 | 0.44 | 0.41 |
| **D Cys%** | 0.37 | 0.34 | 0.30 |
| **D Me+Cys %** | 0.91 | 0.78 | 0.71 |
| **D Lys %** | 1.26 | 1.08 | 0.90 |
| **D His %** | 0.56 | 0.49 | 0.42 |
| **D Tryp %** | 0.28 | 0.25 | 0.21 |
| **D Thr %** | 0.82 | 0.71 | 0.59 |
| **D Arg %** | 1.48 | 1.28 | 1.04 |
| **D Iso %** | 0.93 | 0.81 | 0.67 |
| **D Leu %** | 1.66 | 1.45 | 1.21 |
| **D Val %** | 1.01 | 0.89 | 0.75 |
| **D Gly %** | 0.92 | 0.81 | 0.69 |
| **D Ser %** | 1.06 | 0.94 | 0.79 |
| **Phytate P %** | 0.24 | 0.23 | 0.21 |
| **Na %** | 0.20 | 0.20 | 0.20 |
| **Cl %** | 0.28 | 0.29 | 0.29 |
| **K %** | 1.04 | 0.91 | 0.76 |
| **Linoleic acid %** | 1.39 | 1.75 | 2.16 |
| **Na+K-Cl** | 273.86 | 240.17 | 199.04 |
| **DUA** | 238.56 | 208.67 | 186.01 |
| **Sulphur%** | 0.24 | 0.21 | 0.19 |
| **Magnesium** | 0.16 | 0.15 | 0.13 |
| **Betaine** | 0.71 | 0.80 | 0.90 |
| **Choline** | 1,506.50 | 1,361.46 | 1,189.47 |
| **Poult ME MJ/kg** | 12.55 | 12.97 | 13.39 |
| **Poult NE Kcal/kg** | 1,946.80 | 2,057.64 | 2,176.96 |
| **Gly+ser** | 2.20 | 1.95 | 1.64 |

**Supplementary Table 2**. Average *Salmonella* counts isolated from faecal samples.

| **Study day** | **T1 (CFU/g)** | **T2 (CFU/g)** | **T3 (CFU/g)** | **T4 (CFU/g)** | **T5 (CFU/g)** | **T6 (CFU/g)** |
| --- | --- | --- | --- | --- | --- | --- |
| **D6** | 0.00 | 0.00 | 7.39 x 10^4^ | 0.00 | 0.00 | 8.75 x 10^1^ |
| **D7** | 0.00 | 0.00 | 4.53 x 10^5^ | 3.16 x 10^5^ | 4.83 x 10^5^ | 5.10 x 10^4^ |
| **D8** | 0.00 | 0.00 | 9.15 x 10^5^ | 2.77 x 10^5^ | 1.31 x 10^5^ | 7.04 x 10^4^ |
| **D9** | 0.00 | 0.00 | 8.40 x 10^5^ | 3.59 x 10^5^ | 7.40 x 10^5^ | 6.33 x 10^4^ |
| **D10** | 0.00 | 0.00 | 1.56 x 10^6^ | 2.36 x 10^5^ | 1.50 x 10^5^ | 1.05 x 10^4^ |
| **D14** | 0.00 | 0.00 | 4.96E x 10^6^ | 4.96 x 10^5^ | 3.05 x 10^5^ | 3.23E x 10^4^ |
| **D28** | 0.00 | 0.00 | 5.73 x 10^4^ | 2.08 x 10^2^ | 8.54 x 10^2^ | 6.28 x 10^2^ |
| **D42** | 0.00 | 0.00 | 3.06 x 10^4^ | 0.00 | 6.46 x 10^2^ | 1.88 x 10^2^ |
